# Analectic

**Published:** 1829

**Authors:** 


### MISCELLANEOUS INTELLIGENCE.

### *ANALECTIC, ANALYTICAL, AND ORIGINAL.*

### I. Analectic.

1. *Amputation of the neck of the Uterus.**—As operations on the uterus appear to be growing very fashionable, perhaps it would be useful to give some account of the mode of proceeding adopted by Lisfranc.

The patient was a woman 49 years of age, and the operation performed on the 4th of July, in the following manner. The neck of the uterus being too much enlarged to allow of its being seized at the bottom of the vagina, it was necessary to draw it down to the orificium externum, in order to determine the limits of the disease. This preparatory step was, however, very difficult, in consequence of the tumour being fungous and softened, whilst there only existed a kind of band above, where the tissue of the organ was so firm as to admit of the necessary traction. This difficulty was surmounted by M. Lisfranc introducing the fore-finger of the left hand so deeply, that its point passed the limits of the fungus, and then carrying forwards upon it a hook, (*une aizique*) guarded by lying with its flat side on the finger. Having reached the sound part of the uterus, the hook was turned round and impacted in its substance, by pressure with the end of the finger. Many other hooks were introduced in the same manner, and the diseased mass drawn down, by their means, to the labia externa, when a pair of forceps, (pince de museux) was applied, and several of the hooks were withdrawn. The tumour being retained in this position by assistants, the operator proceeded to ascertain the exact limits of the disease, and the point of attachment of the membrane of the vagina, rather by means of the finger than the eye. The labia were then separated as widely as possible, and the band of sound parts cut across by a bistoury with a very concave cutting edge, the section being made from behind towards the front, and care being taken to prevent any obliquity.

These were the principal features of the operation, which we give for the sake of recording the *how*, and not of discussing the *why*. The most difficult and embarrassing part of the proceeding, was getting the tumour to the mouth of the vagina, a difficulty that is sometimes completely insuperable. In a case of polypus uteri at St. George's, under the care of Mr. Brodie, the body was so large

*La Clinique.

that the efforts to draw it down were ineffectual, and in consequence abandoned. In a case which occurred to M. Dupuytren, the traction was also ineffectual, until the perineum was divided! In the case of Mr. Brodie's to which we have alluded, the operation was concluded by throwing a ligature, by means of the double canula, round the neck of the polypus, in situ. The ligature was tightened de die in diem, and the tumour sloughed off at the end of a week. The operation was accomplished in a very few minutes, occasioned but trifling pain, and produced no subsequent inconvenience whatever.

*Med. Chir. Rev. Oct.* 1828.

---

2. *On the treatment of Cancerous Affections of the Cervix Uteri, and especially on amputation of that part, by* M. LISFRANC; *reported by* M. AVENIL.—In a late number of our Parisian contemporary, [The REVUE MEDICALE] there is a long Memoir on this important subject, and ten cases are detailed of this formidable operation. We shall, as usual, greatly compress the matter of the Memoir before us.

M. Lisfranc commences with some observations on the accidents which may or do supervene on the operation of excision. The first of these is hæmorrhage. This accident, he thinks is too much dreaded by surgeons, and the pluggings of the vagina to which they have recourse for its suppression, leads to inflammation and worse consequences than the hæmorrhage. On these accounts, he always permits a copious flow of blood from the excised parts.

"When a woman has not been exhausted by preceding bleedings—when she is not extremely weak—when the hæmorrhage does not appear to proceed from large vessels, but, on the contrary, is so slow that there is not more than twelve or fourteen ounces lost in the first two or three hours—then the hæmorrhage should not cause any alarm in the surgeon's mind—nor should the process of plugging the vagina be employed."

M. Lisfranc here describes a series of phenomena which take place when a clot of blood forms in the passage, and produces the same effects as plugging. Among these are, pain in the loins, epigastrium, and region of the uterus, sense of distension, hiccup, nausea, vomiting. When this last occurs, the clot is generally driven away, and all the symptoms are immediately relieved. When the abovementioned phenomena, however, continue without this natural relief, M. Lisfranc introduces his fore-finger, breaks away the coagulum, and re-excites the hæmorrhage, when the strength of the pulse, and the state of the patient appear to warrant the renewed loss. When the mechanical removal of the clot does not re-produce the hæmorrhage, he throws up warm water, which generally has the desired effect. After the storm has ceased, it often happens, that two or three times in the course of the day, sickness and vomiting recur. On this account, the patients in LA PITIE are always operated on at 9 o'clock in the morning, and all the troublesome symptons are usually over by five or six in the afternoon. When the spontaneous or artificially excited hæmorrhage does not relieve the symptoms, M.

Lisfranc prefers bleeding from the arm to leeching the groins or labia. He thinks the former acts more beneficially than the latter. Low diet, diluents, lavements, emollient fomentations, &c. are had recourse to, as auxiliaries, if the foregoing measures fail. Generally a most delightful calm succeeds the first effects of the operation. The dreadful disease removed, the patient tastes a refreshing sleep the first night, to which she had been long a stranger. There is a marked change in the MORALE as well as in the PHYSIQUE. Her gaiety is renewed, and her gratitude to the man who has delivered her from such torments, is expressed with unbounded energy. A complete convalescence is often observed in two or three days after the operation. But great care, absolute repose, and low diet, should be insisted on for eight or ten days, even where no bad symptom appears.

In two cases, where nothing particular occurred during the first two or three days, and where there was no appearance of hysteritis or peritonitis, nor any sign of them on dissection, the patients were suddenly seized with pain in the head, dizziness, weakness, and quickness of pulse, great prostration of strength—death. In these cases M. Lisfranc asks, if the fatal issue did not depend on the absorption of cancerous or poisonous matter from the wound, as there was nothing, on dissection, to account for their death?

When the cicatrization of the wound does not proceed in a favorable manner, M. Lisfranc employs injections containing solutions of the chloride of soda, weak at first, and gradually increased in strength. If this fails, and fungous excrescences are detected on the surface of the wound, he employs the cautery. The medium period required for cicatrization, is six weeks or two months. Great attention should be paid to the general health for a long time after recovery.

CASES.—*Case* 1. Constantia Franck, aged 33 years, became affected with pains in the uterine region, after great mental anxiety and reverses of fortune. Towards the close of 1826, she left Brussels, and came to Paris to consult M. Lisfranc. He detected an incipient cancer of the cervix uteri; and some ulcerations had already taken place around the os uteri. The pains were lancinating and constant, with fetid excoriating discharge. Two months of antiphlogistic measures produced no amelioration of the symptoms, and she entered LA PITIE to undergo the operation, on the 3d of January 1827. Three weeks had elapsed since the last consultation, and the disease was found to have made progress. The operation was performed according to M. Lisfranc's usual method (already described in this Journal) and the patient suffered little. In one hour after the operation, the hæmorrhage became very abundant. M. Lisfranc sat by the bed-side, and did not plug the vagina. In two hours, the bleeding lessened, and a clot formed. Colicky pains next occurred, and a fit of vomiting expelled the clot. The pains immediately ceased. A slight syncope supervened, but the pulse did not sink much, and the plugging was avoided. In the same evening there were symptoms of excitement that rose into strong re-action, requiring venesection to six ounces. We need not pursue the diurnal details. The nitrate of silver was applied on the 29th of January. On the 24th the patient left the hospital cured.

*Case* 2. Ellen Loos, aged 30, the mother of six children, became affected, after two abortions, with lancinating pains towards the groins, loins, thighs, and region of the uterus. The menses got irregular, and, for nine months previous to her entrance into LA PITIE, she had considerable discharge from the vagina. Her mother died of cancer of the uterus. The patient entered the hospital on the 20th January, 1827. She had had 300 leeches applied in the course of three months at two other hospitals. On examination, the cervix uteri presented considerable tumefaction, and deep ulcerations, from which a fetid sanious pus issued. The operation was deemed indispensible, and was performed on the 23d of the same month. The uterus in this case could not be drawn as low down as usual, although it was in a state of slight prolapsus. Five lines in depth of the cervix uteri was removed. Considerable hæmorrhage ensued, and most of the symptoms described in the preamble of this paper. But the patient ultimately did well, and by the 18th of March the cure was complete.

*Case* 3. Augustine Conder, aged 25, became affected with lancinating pains in the uterine region shortly after an abortion, which she had experienced eight months before she entered the hospital, which was on the 15th February, 1827. The cervix uteri presented a large ulceration, especially on the anterior lip of the os uteri, with reverted edges, and an indurated state of the neighboring parts. The uterus itself was double its natural size. The operation was performed on the 19th, and offered nothing worthy of remark. Very little pain was experienced while the cervix was incised—the whole of the deceased part was removed—and there was scarcely any blood lost. Some smart hæmorrhages afterwards supervened, but they did not require the plug. There were considerable pains, and symptoms of constitutional disturbance in the course of the first few days, requiring several bleedings, and all the usual antiphlogistic measures. On the 1st of March the menses appeared, and flowed for two days, and on the 14th of the same month, she left the hospital completely cured.

*Case* 4. Harriet Cuiret, aged 22, had never borne children. Had experienced, for more than a year, lancinating pains in the region of the uterus, with an abundant leucorrhœa. The menses became irregular, and for the three preceding months, had scarcely been seen. She entered LA PITIE on the 30th of January, 1827, for another complaint, during the cure of which she disclosed the more important malady. The cervix uteri was found to be tumefied, indurated, profoundly ulcerated. The operation was performed on the 6th of March, as M. Lisfranc was of opinion there was no time to lose. There was great difficulty experienced in the resection, on account of the narrowness of the parts. The surgeon was obliged to use considerable force in separating the parietes of the vagina, and perform the section of the cervix in the interior of the vulvo-uterine canal. There was little blood lost at the time; but great hæmorrhage succeeded, and was arrested by syncope, and the formation of a clot. The clot was afterwards expelled by vomiting—and these formations and expulsions were four or five times reiterated alternately. In one of these hæmorrhages there was a loss of more than 20 ounces from the wound. On the 7th April a copious menstruation took place; and on the 11th of the same month, she was seized with an intermittent fever, which ceded to the quinine. On the 27th July she was discharged perfectly cured.

*Case* 5. Claudine Deschampts, aged 35, was delivered safely of a child in December, 1826, soon after which the menses were suddenly suppressed by a violent mental emotion. Uterine pains, of a lancinating character next succeeded, and, on the 6th March, 1827, she entered La Pitie. Large vegetations were discovered on the cervix uteri, and similar excrescences were seen about the verge of the anus. M. Lisfranc thought them venereal, and prescribed mercury, under the influence of which, the external vegetations completely disappeared; but the others continued unaltered. The cervix uteri now became tumefied, indurated, and, in fact, the malady made such progress, that M. Lisfranc decided on an operation. It was performed on the 28th May, at nine in the morning. The hæmorrhage was trifling till she was carried to bed, when it became considerable, and syncope was twice imminent, accompanied by chills. A large clot formed in the vagina. At eleven o'clock that night, nausea, hiccup, vomitings came on, and the clot was instantly expelled; when all the foregoing symptoms ceased. They again recurred on the formation of a clot, and again the clot was expelled. There were some difficulties in the management of the case, but they were overcome; and, on the 30th June, a profuse menstruation came on. In five weeks the cicatrization was completed.

*Case* 6. Madame R——, 42 years, perceived in the year 1823, after some violent moral afflictions, severe pains in the loins and groins, accompanied by leucorrhœa, for which she underwent remedial treatment, without relief for two years. She then consulted M. Serres in Paris, who found the os tincæ surrounded with slight ulcerations, which were touched with caustic, and this process produced much relief. Eighteen months afterwards she was induced again to return to Paris, and M. Serres found that extensive ulcerations obtained around the mouth of the uterus. He proposed ablation of the cervix, and M. Lisfranc was summoned. This gentleman found deep ulcerations, surrounded with stony hardness, and attended with fetid discharge. On the 26th April the operation was performed, without any thing particular occurring afterwards; and on the 27th June she was pronounced to be completely cured.

*Case* 7. Rose Rudell aged 27, had experienced for the five preceding years, and without any known cause, severe pains in the loins, copious leucorrhœa, shooting pains in the region of the uterus, irregular menstruation. These symptoms were accompanied by general derangement of the health, and a yellowish tint of the skin. She entered the Hospice de Parfectionnement in the beginning of October, 1827, at the time when M. Lisfranc was on duty there. On examination, by means of the speculum, he found ulcerations and induration of the cervix. On the 19th November the cervix was completely removed, and it was evident from the ulcerated state of the parts, that the operation was not practised an hour too soon. The usual symptoms occurred after the operation, and she left the hospital completely cured, on the 31st of December.

We do not deem it necessary to pursue the analysis of the remaining cases. Sufficient have been adduced to show the ordinary symptoms that precede the operation, and the accidents that follow it. In the course of the last four years M. Lisfranc has performed this formidable operation, in the hospital and private practice, thirty-six times. In thirty-three cases the operation was crowned with success. Three of the patients died:—one on the 18th day after the operation—apparently from an affection of the liver, and an encephaloid disease behind the uterus—the second after three months of good health, when the uterine disease returned—the third died from an affection of the spleen, not suspected to exist at the time of the operation. The reporter, M. Avenil, witnessed three other operations of this kind in Paris,—one by Dr. Ricord, one by Professor Recamier, and one by Dr. Hutin. In two of these three cases complete success followed, and the patients now enjoy excellent health. One of them became pregnant in the course of a month after the operation, and was safely delivered. She has since that period borne twins.

When we consider the fatal nature of this disease—the dreadful and lingering torments by which the patients are consumed—the comparative facility of this operation, and the almost insuperable objections to a total ablation of the uterus, we cannot but regret that English surgeons should be behind their continental brethren in courageously affording relief to, their suffering country-women, upon such trying occasions. We hope the present Memoir will induce them to take early means for ascertaining the approaches of uterine disease, and that the success of M. Lisfranc and others, will stimulate them to operate before the progress of the malady renders the operation terrible, and the result precarious.—*Ibid. Jan.* 1829.

---

3.—*Treatment of Varicose Ulcers.—Case* 1—*Division of the Vena Saphena.*—A soldier, 32 years of age, of a good constitution, received a burn in the lower part of the leg, nearly two years before his reception at La Pitie. At this time there was an ulcer, the size of the palm of the hand, and several ramifications of varicose veins were perceived upon the leg. The vena saphena was divided by M.

Lisfranc, below the knee; no sympton whatever of phlebitis succeeded, and in eighteen days the wound had healed. In twenty-four days after the operation the patient was discharged completely cured. He now returned to his usual occupations, retaining no bandage or compression on the leg, the cicatrix got abraded, and the patient re-entered La Pitie on the first of August. The ulcer was now not so large as before, and a subcutaneous vein, about as thick as a crow quill had appeared before and below the cicatrix made by the former operation. Simple applications, and the horizontal posture, effected a cure in eighteen days, when the patient was a second time dismissed, with careful injunctions to wear a laced stocking.

*Case 2. Ulcer of the Leg.—Excision of part of the Vena Saphena.*—Charles Perlot, ætatis 52, a house painter, of only an indifferent constitution, was admitted into La Pitie, with an ulcer of twelve months standing, a little above the internal maleolus. The sore was as large as the palm of the hand, and the treatment consisted in the application of emollients and poultices. On the 23d of June, a fortnight after his admission, part of the saphena externa was excised below the knee. On the day of the operation, he required a bleeding from the arm, and, complaining on the morrow of pain in the limb, sixty leeches were applied on the inside of the thigh, followed on the 25th, by a relay of forty more. On the 26th there was a third application of leeches, and the saphena, on the 27th, presented the symptoms of inflammation of its coats, viz. tension, swelling, and tenderness on pressure. Fifty leeches were placed on the upper part of the thigh, but these not sufficing, the same number was twice repeated. The symptoms of phlebitic inflammation subsided, an abscess formed about the middle of the thigh, it was opened, and a wine-glass full of pus escaped. From this time no untoward accident occurred; the wound was healed at the end of five weeks, but the ulcer continued open for about two months, when a laced stocking was applied, and the patient dismissed.

In the first of these cases, *division* of the vein was had recourse to, in the second, *excision*, and mark the results. In the former no local irritation was excited; in the latter, phlebitis was the consequence, and the patient had a narrow escape with his life. Division, with a valvular opening in the skin, is, generally speaking, a safe operation; excision, or ligature, certainly is not. It is but the other day that a patient was lost at Bartholomew's Hospital, by cutting out a portion of a varicose vein, and the experience of the leading men in the profession is almost universally opposed to the practice. The opinions of Sir Astley Cooper, on the subject, are expressed in his usual frank and energetic manner.

"It was formerly the practice, when the veins were in a varicose state, to tie and divide them. This plan is still pursued by many surgeons; but it is one that I have deprecated in my lectures, in this Theatre, for the last eight or nine years; it is very injudicious, and fraught with great danger, therefore let me exhort you never to adopt it. I have seen this operation prove fatal in several instances, in these hospitals; therefore I was induced to say, that it must not be performed. A gentleman at Nottingham informed me, that he had tied the vena saphena, for a varicose state of the veins of the leg of a young farmer, in other respects healthy, and the operation proved fatal. The same lamentable catastrophe occurred to a most respectable practitioner at Brentford; and this gentleman told me that he would not again perform the operation for the world. If I were to tell you all the cases in which I have known it terminate fatally, I should recount at least eight. Another overwhelming objection to the operation is, that when it does not prove fatal, its ultimate effects are useless. If I were asked which of the following operations I would rather have peformed upon myself, viz. the saphena major vein, or the femoral artery tied, *I certainly should choose the latter!* When an artery is tied, the inflammation is confined to the neighborhood of the ligature; but in a vein, it is very extensive, the vessel becomes exceedingly distended, the inflammation uncommonly severe, either extensive suppuration or mortification ensue, and death is the result."—*Sir A. Cooper's Lect. by Tyrrel, Vol. I.*

The worst of it is, that even where the operation does not kill, it often fails to cure. Again and again have we witnessed division of the vein followed by relief, indeed, a cure, for the time, but quite ineffectual in its ultimate results. The patient returns with the leg as bad as ever, other veins having arisen to occupy the place, or we should say, the office of the one that is obliterated by the knife. Is the surgeon to continue cutting vessel after vessel, running risk after risk, or trust to compression, and palliate what it is not in his power to prevent? We should decide in favour of the latter proposition, and apply the laced stocking, or equable bandaging. Bandages, we think, are the better of the two, but seldom, in the upper classes, admit of being worn. The patent silk bandage, (we really are ignorant of its exact *scientific* nomenclature) is at present much in fashion, and may be had at a mercer's in Oxford street. The fact, however, is, that its great elasticity, for which it has been praised, is its worst and most objectionable quality, as it *gives* far too much to make steady compression. This, in connexion with its comparatively high price, will forever prevent its coming into general use. The lower classes cannot afford it; for the higher, it has most of the inconveniences, and not all the good properties of the cotton roller.

The ulcers which depend upon varicose veins, or varicose ulcers, as they are frequently, and not very properly called, are generally healed by repose, common stimulating applications, as the solution of the nitrate of silver, &c. and especially bandaging and strapping. We say they are healed, for a pellicle of skin will form upon the surface, in nine cases out of ten, by the above means. Though covered, however, with ashes, and extinguished to the view, the fire is lurking and smouldering below, ready to burst forth on the least exciting cause, and rage as extensively and fiercely as before. It is really astonishiug how quickly these varicose ulcers will skin over, and how quickly again they will break out afresh, when the patient resumes his usual avocations. Careful and continued bandaging, with standing, about as little as possible, are all the prophylactics that the surgeon possesses, or the patient can trust to. We shall not advert to the employment of blisters and irritating applications in the neighborhood of the varicose vessels. They may be regarded rather as matters of curiosity than practical utility.—*Ibid.*

---

4.—*Lithontripteur.*—Mr. Zanabi Pecchioli, an eminent young surgeon, charged by the Grand Duke of Tuscany to observe the actual state of surgery in various countries, has made a great improvement on, or rather he has added a new and important principle to, the lithontriptic instruments invented by Messrs. Leroyd d'Etoile, Civiale, Amusat, Hourteloupe, &c. &c. We have had a recent opportunity of examining Mr. Pecchioli's instrument, and seeing him work it on different calculi—not, of course, in the living body. We would say that its superiority over the instruments of the gentlemen above named, is threefold. In the first place, it combines the principles of each of the others, the drills and other parts of their machinery being rendered completely available in Mr. P's. apparatus. In the second place, the spring, or ressort, by which the drill or perforator is made to bear on the calculus, and which cannot in the other instruments, be made to vary in force, is superseded by the construction of the pulley, which enables the operator to modify, vary, augment, or diminish, at pleasure, the force used—and that by his own hand. This we conceive to be a very important improvement. But the third modification is the most important of all.—The perforator or drill, in Mr. P's. lithontripteur, can, at any period of the operation, be converted into a kind of trephine, varying in the diameter of its circular movements from the smallest circle up to one of 18 lines in diameter, at the operator's will—and thus becoming capable of grinding down the calculus by a series of gyrations equal in extent to the grasp of the pincers or tenacula, instead of boring holes, and shifting the instrument for each perforation. By this operation, a considerable portion of stone may be ground down by a single sitting; and the danger of large and irregular fragments being scattered about in the bladder, when the calculus is broken after many perforations, according to the methods of Leroy d'Etoile and Civiale, is avoided.

Sir Astley Cooper, Mr. Travers, Mr. Key, and many other distinguished surgeons, have compared Mr. Pecchioli's apparatus with that of M. Civiale's; and, without vouching for the general success of the lithontriptic process, they have no hesitation in acknowledging the great ingenuity of Mr. P's. instruments.

P. S.—When Sir Astley Cooper was in Paris last month, he went to see M. Civiale operate with the lithontripteur. The subject was not one of the best for any operation. M. Civiale threw in half a pint of tepid water—introduced the lithontriptor with the greatest ease, seized the stone, drilled it, and then crushed it, all in the space of about seven or eight minutes. The operation over, the man discharged the water which had been injected, quite turbid with the sawings of the stone, and when poured off, disclosing numerous fragments that had come away with the first evacuation of the bladder after the operation. Sir Astley was quite astonished at the facility with which the whole was performed by M. Civiale. We apprehend that this operation *must* become popular in the hands of a *few* expert surgeons; but we do not suppose it will ever become general among surgeons at large.—*Ibid.*

---

5.—*Action of the Colchicum on the Urine.*—Professor Chelius, of Heidleberg, has been making a number of experiments on the urine of those who take colchicum for different complaints, and has discovered a very curious and uniform effect to result from this powerful medicine—namely a great increase of uric acid in the urine—and consequently, a great evacuation of it from the system at large.—Thus a man, who had rheumatic inflammation and swelling of several joints, was put upon the use of colchicum, his urine being first examined, and found to contain 0,069 of uric acid, free or combined with ammonia. In four days after the colchicum was commenced, and that in moderate doses, it rose from 69 to 76. On the eighth day it was 91, and on the 12th day it was 102. In short, in the course of a fortnight, the quantity of uric acid was doubled. Experiments were made on a great number of individuals, chiefly laboring under gouty, rheumatic, and neuralgic affections, and all with the same result—a great increase of the uric acid. Professor Chelius thinks, that this operation of the colchicum may lead to some elucidation of the specific efficacy of the medicine in various diseases, and perhaps may throw some light on the nature of those diseases themselves. The Professor appears to employ the colchicum with great success, in neuralgiæ of the face, in sciatica, rheumatic ophthalmia, dropsy of the joints, and in certain paralytic affections of the lower extremities connected with the arthritic diathesis. We would recommend the medical practitioners of this country to note the effects of colchicum on the urine; as the increase of uric acid may lead to the useful employment of this remedy in some diseases to which it is not now applied.—*Ibid.*

---

6.—*New mode of treating Tænia, discovered by* Dr. Schmidt, *of Berlin, aud described by* M. Caspar, *by order of the Prussian Government.*—On the 14th of October, 1823, Dr. C. A. Schmidt, of Berlin, announced to the minister of public instruction, and

Medical affairs of Prussia, that he had, twenty years since, discovered an infallible remedy for the tape-worm, and was desirous of selling his secret to government. The minister directed Dr. Natorp to make trials with the practice, and, in a report made the 25th of July, 1824, this physician pronounced the treatment of Dr. Schmidt to be excellent; that it was adapted to the most feeble constitutions; brought away the tape-worm within twenty-four hours at furthest; called for no previous preparation, and caused no more exhaustion than a common purgative. Messrs. Kluge and Nureman, physicians to the hospital of La Charitie, at Berlin, were commissioned to repeat these experiments. In the report made by these gentlemen, on the 31st of October, 1826, they express themselves thus:—"The method of M. Schmidt never failed in its effects when the presence of the tænia was established, and those cases wherein it failed to produce evacuation of a worm, the existence of this was regarded as doubtful. At the same time, the treatment is prompt, without danger or exhaustion, and the worm is expelled entire and alive." In consequence of the report made him by the minister, the king of Prussia granted Dr. Schmidt, on the 31st of March, 1827, a pension of one hundred and fifty dollars for the publication of his remedy, which he described as follows:—First day the patient takes in the morning, fasting, two spoonsful of the following preparation, and the dose is to be repeated every two hours till seven o'clock in the evening.

No. 1.—Take powder of valerian root, six drachms; senæ leaves, two drachms; make into an infusion of six ounces, to which add sulphate of soda, three drachms; syrup of manna, two ounces; oil of tansy with sugar,* (oleo-saccharum de tanaisie) two drachms. Mix.

The patient drinks coffee made very sweet, and without milk; at noon plain soup with some morsels of herring, and the roe of this fish; at eight o'clock in the evening a salad made of herrings, raw ham sliced, one onion, with a considerable quantity of oil and sugar. The patient most commonly expels, even on the first day, some portion of the tape worm. In two cases M. Schmidt has even seen the worm evacuated whole from this preliminary treatment alone. The second day the patient takes every hour, beginning at six o'clock in the morning, the following pills:—

No. 2.—Assafœtida, dogs-grass, (chiendent,) each three drachms; powdered gum gamboge, rhubarb and jalap, each two drachms; powdered digitalis, ipecacuanha, golden sulphuret of antimony, each twelve grains; calomel, two scruples; oils of tansy† and anis, each fifteen drops. To be made into a mass, and divided into pills of two grains each, which are to be kept in a well stopped bottle.

---

* This is prepared by adding twenty-four drops of oil of tansey to one ounce of loaf sugar.

† Obtatned by distillation of the fresh plant.

These pills are taken with a tea-spoonful of syrup. Half an hour after the first dose, the patient takes a table-spoonful of castor oil, and through the day drinks freely of coffee well sweetened. In most instances the worm is evacuated about two o'clock in the afternoon, in which case the pills are to be stopped; but where, on the contrary, only fragments of the tænia are passed, the pills are to be continued, along with which a spoonful of castor oil with sugar is to be given from time to time: the treatment is to be discontinued whenever the evacuations cease to contain any of the worm. At noon the patient takes nothing but broth, and in the evening some soup with fresh butter and sugar. To make sure that, (to use the author's mode of expression,) no more of *the nest of the tænia* remains, the patient may still take some of the pills in the morning. To prevent relapses, the patient ought occasionally to eat herring sallad and horse-radish with vinegar and sugar; or, otherwise, he should continue for some time to take, about every eight days, one or more doses of the pills. When the treatment is finished, the patient is allowed to eat gruel, young meats, chicken, pigeon, the yolk of eggs, good wine in small quantity, and some kind of bitters.

Where it is not certain that tape worm exists, the following method of ascertaining its presence or absence is adopted. The patient eats herring sallad in the evening, and drinks freely of sugar and water; the following morning he uses the following powder in syrup: take powdered jalap, gr. xv. wormseed, gr. x. gum gamboge, calomel, aa. gr. vi. oil of tansy, (oleo-saccharum) ʒi. After this powder, the patient drinks coffee made very sweet, or very fat broth. The powder produces copious alvine evacuations, and if the patient be affected with tænia, joints of it, or sometimes the entire worm will appear in the stools. In this last case, the pills are to be followed up by the treatment No. 2, for the purpose of establishing a complete cure, should there be more than one tænia.

Dr. Schmidt does not employ his treatment during pregnancy, nor immediately before or after the menstrual period, nor with individuals affected with inflammations, phthisis, marasmus, hæmorrhoids, hæmoptysis, or senile debility.

Of one hundred and sixty-six persons cured of tænia by Doctor Schmidt, but fifteen were men, twenty had but one tænia, and all the others more, one evacuated as many as seventeen. After the publication of Dr. Schmidt's treatment, the experiments were repeated at the Hospital of La Charitie, in Berlin, with constant success, as shown by the six cases which close the memoir.—*Archives Generales, January,* 1829, *from Hufeland's Journal, August,* 1828.

---

7.—*Neuralgia Facialis.*—The *Osservatore Medico di Napoli,* No. 18, contains an account by Dr. Campagno, of a case of neuralgia facialis which was cured by the vinous tincture of colchicum. A multitude of remedies had been previously tried without effect.

*Am. Jour. Med. Sciences.*

8.—*Dysentery.*—Dysentery has recently prevailed to a considerable extent in the Edinburgh Infirmary. The treatment adopted by Dr. Christison, "consisted at the commencement in the liberal use of opium, preceded in some instances by the free application of leeches to the lower part of the belly, and frequently accompanied with the application of large blisters, and with the use of the warm bath. If the stage was passed during which feculent matter was discharged, and the evacuations had become muco-sanguinolent or sero-sanguinolent, I usually directed the application of the leeches to be immediately followed by doses of pure opium, of such magnitude and frequency as were found necessary to check the unremitting diarrhœa and tormina; and sometimes the desired effect was not procured till the patient was pretty strongly affected by the narcotic action of the drug. In urgent cases twenty or twenty-four grains in the twenty-four hours were sometimes necessary from the very beginning; in the slighter cases four or six grains were sufficient. When an impression was once made on the discharges, it was maintained by doses of two or three grains repeated according to circumstances; and frequently the exhibition of opium by the mouth was conjoined with its employment in the form of suppository. I never but once found this plan to fail in checking the discharge of blood in twenty-four or forty-eight hours, if the patient was seen within three or four days; but the blood often reappeared abundantly in the stools, if the opium was intermitted on account of its causing too complete constipation. After the hæmorrhage was permanently checked, the frequent thin feculent stools continued many days, sometimes many weeks, indicating, it is to be presumed, the existence of ulceration, which consequently must have taken place at a very early period of the disease. The opium, it is worthy of remark, rarely caused sickness or dry tongue. In cases in which the stools continued long thin, and with a tendency to be tinged now and then with blood, an opportunity occurred for trying various remedies which have been supposed to be useful in this stage by accelerating the cicatrization of the ulcers. But I cannot say that any of them appeared to be of use, unless opium was combined with it in such quantity as to be itself a powerful agent. The acetate of lead was perhaps an exception; it certainly rendered the stomach less irritable, in the few cases in which opium alone was rejected by vomiting; and although I had too few opportunities of trying it in idiopathic dysentery, the experience I have had in the Fever Hospital and Infirmary fever-wards of its good effects in the chronic dysentery which is sometimes left after fever, induces me to think that its alledged virtues in the chronic stage of idiopathic dysentery have not been exaggerated.—Neither ipecacuan, nor nitric acid, nor calomel administered so as to affect the mouth, appeared to me materially useful. I have had but one opportunity of trying the effect of calomel in scruple doses upon the early stage of the disease. It was given on the fourth day with marked advantage, certainly, and was repeated next day with equally good effect. But ulceration had evidently taken place before the patient came under my charge; and although the acute symptoms were checked, yet, as the patient was an emaciated subject transferred from the surgical wards with a recently opened, extensive, chronic abscess, he sunk under the exhausting purulent discharge from the bowels and abscess together. The only other patients I lost were two in number,—one an old man of ninety, who entered the hospital on the eighth day of his illness in a state of extreme exhaustion, so that, although the stools were checked, he died two days afterwards,—the other, a young woman, who immediately after coming out of an exhausting and tedious attack of continued fever with marked enteritic symptoms, was seized with dysentery in its worst form, and died on the tenth day without experiencing any relief from treatment beyond the allaying of pain."—*Ed. Med. and Surg. Jour. January*, 1829.—*Ibid.*

---

9.—*Complete Amaurosis cured by the application of Leeches to the Nasal Fossæ.*—Dr. Guepinet of Landrecies, relates in the *Annales de la Medicine Physiologique, Vol. X.* the case of a child aged five years, who was suddenly attacked with complete amaurosis, without any known cause. The disease resisted the usual remedies for near two months and a half, when Dr. G. being consulted, he advised the application of leeches to the nassal fossæ. The day after the application the child was able to see a little; thus encouraged, Dr. G. had one or two leeches applied daily for a week, at the end of which period the child's sight was entirely re-established.

*Am. Jour. Med. Sciences.*

---

10.—*Remarkable Superiority of amputatingby one Incision through the Integuments and Muscles.*—This great improvement in the mode of amputating, which was formerly recommended by Louis, and unaccountably abandoned, is practised and highly recommended by all the present surgeons of the Hotel Dieu, M. Dupuytren, M. Breschet, and M. Sanson. In thighs removed by the two former, and an arm by the latter, no tourniquet was employed in either case. The limb was grasped by an assistant, and pressure made on the principal artery. A single incision cut through skin and muscles down to the bone, and a retraction of the skin and muscles not inserted in the bone was effected to the extent of three inches. The first retraction having been completed, the muscles attached to the bone were cut through by a scalpel, on a level with the others, and the bone sawed as usual. The stumps in all were remarkably fine, and the strength of the bone was more than sufficiently recovered.

After each operation, the surgeons respectively explained their reasons for preferring this procedure, whose expediency was proved by the result of every case in which it has been tried of late. To say nothing of the discarding of the tourniquet, which enables the operator to effect a retraction of the muscles to a greater extent than under its use; the old method of preserving the skin by cutting it up from the muscles to which it naturally adheres, was a considerable impediment to union by the first intention.—*Lond. Med. and Phys. Journal.*

---

11.—*On Blistering Infants.*—The melancholy consequences which frequently arise from the application of blisters to young children, renders every suggestion which is probable may prevent them of great importance. We find that an hour or an hour and a half is a sufficient time for a blister to remain upon a child, and although at the expiration of that time no vesication is apparent, yet, if the part be covered with any mild dressing or a poultice, a sufficient degree of irritation will be observed in a few hours' time, in fact quite sufficient for the peculiarly delicate and susceptible constitution of children. When the emplast. cantharides has been employed in this manner, we have never witnessed any alarming result.—*Lon. Med. and Surg. Journal.*

---

12.—*Neuralgia.*—Dr. Wilson the talented author of a work on West India Fevers, reviewed in a former number of this Journal, has transmitted to us some observations on the dreadful case of Neuralgia lately published for consultation, in No. XVIII. of this Journal, page 435. We shall take the liberty of quoting a passage from Dr. Wilson's letter, as it contains some practical facts of interest and utility.

"In three cases of neuralgic disease lately under my care, a cure was effected, by the combination of calomel, opium, and oil of turpentine. In my memoirs of West India Fever, I there stated my belief that the oil of turpentine not only accelerated the action of mercury, but also, in certain cases, increased its curative effects. In the neuralgic cases to which I allude, I gave the turpentine chiefly with the view of speedily inducing the specific action of the mercury. I am now, however, inclined to believe that the turpentine operated beneficially in itself. The following is the mode of administration which I have employed in the three neuralgic cases above-mentioned.

A pill, containing from two to four grains of calomel, and one or two grains of opium, was given each night at bed-time, and, next morning, one or two drachms of oil of turpentine, mixed with a little honey. In each of the three cases, a complete and permanent cure has been effected by this plan, and in a moderate space of time."

*Med. Chir. Review.*

---

13.—*Ascites cured by Pressure.*—Our readers are aware that Sir Gilbert Blane proposed pressure on the head, for the cure of hydrocephalus, and that the plan has been adopted by some others—not, we should suppose, with great success, otherwise we should have heard of it. Anasarca of the legs is every where treated by pressure, in the form of bandages, to promote absorption—or, rather perhaps to force the serous infiltration into another and worse locality. Indeed, we have generally observed, that patients have complained of oppression about the chest, or sense of fulness in the abdomen, when tight bandages were applied to prevent or remove anasarcous swellings. Till the secretions and excretions are set free, there is little permanent relief obtained in dropsy of any kind. The principle of pressure has been applied in the case of ascites, by Dr. Speranza, physician to the Clinical Institution of Parma.

*Case.*—A female had labored for some months under considerable effusion into the cavity of the abdomen, the consequence of peritoneal inflammation, which had succeeded to a laborious accouchment. She entered the Parma Clinical Institution in the month of April, in a very weak condition, being apparently worn down by a slow fever, and lactation beyond her powers. The digestive functions were very much deranged—the urine scanty and turbid—thirst—constipation. The great distension of the abdomen by effusion, prevented any accurate examination of the viscera, as to their state of soundness. There was general emaciation, and a highly cachectic aspect. After a fair trial of diuretics, purgatives, mercurials, and the usual remedies, Dr. Speranza declined to recommend paracentesis abdominis, from a conviction, that this operation is never of any ultimate service; he had recourse, therefore, to the bandage of Monro. Under the influence of the pressure thus produced, the urine became copious, so that, at length, it amounted to *fifteen pints daily!* In the course of three weeks, the abdomen was reduced to its natural size, and there was no further effusion. The sulphate of iron, squills and a nourishing diet contributed to a completion of the cure. She was ultimately discharged from the Hospital in perfect health.

It would be a very curious fact, if proved by subsequent experience, that pressure on a distended abdomen caused an increase of the urinary secretion. We could only account for it in this way:—the pressure augmented the action of the absorbents, or checked the activity of the exhalents, or both—and the blood-vessels being replete with serous fluids, the secretion of the kidneys was augmented and thus the ascites cured. We think the remedy, especially if combined with *diuretic frictions*, might be well worthy of trial.

*Ibid.*

---

14.—*Treatment of Strictures of the Urethra.*—The mode of curing strictures of the urethra by confining a *large* bougie at the anterior part of the obstruction, is still employed with undeviating success in the wards of M. Dupuytren and of M. Breschet at the Hotel Dieu. The mere contact, accurately preserved for eight or ten days, often enables a catheter of the largest size to pass freely where the smallest bougie could not previously penetrate.

"The bougies thus introduced are provided with four very narrow tapes or strings, whereby the are attached to a T-bandage, surrounding the waist and under the scrotum. In their passage they are coiled, at equidistant points, round a ring which is placed over the body of the penis."—*Lon. Med. and Phys. Jour. Jan.* 1829.

---

15.—*Mode of Preserving Specimens of Morbid Anatomy*—By John S. Gaskoin.—Mr. Gaskoin recommends the following means for preserving the appearances of diseased parts:—"Having removed the diseased part from the body, it should be as little handled or dissected as possible, especially when the effects of inflammation, congestion, &c. are to be preserved, as the blood may be pressed from, or disturbed in, the minute vessels. Let the blood which may have escaped from cut vessels, be gently washed off from the surface by a solution of the *muriate of ammonia*, or be absorbed by a soft sponge, lightly applied. The part should then be wrapped with care in old linen, and be so immersed in *one part of a saturated solution of the muriate of ammonia*, (sal ammonia of commerce) and *two of rectified spirit of wine*. After two or three days the linen may be removed, and the part restored to the fluid.

"Should the preparation be large, or from the nature of the disease, contain a large quantity of aqueous fluid, then an additional portion of the muriate of ammonia in powder should be added, to meet the excess of aqueous menstruum.

"The time necessary for maceration will mainly depend upon the size of the part to be preserved; but, generally, from ten to fifteen days will be found to be sufficient, although nothing can be lost by an extension of that time. Being taken from the macerating fluid, it should be again washed in a solution of the muriate of ammonia, then dissected as much as requisite, and be 'put up' at once, *in equal quantities of a saturated solution of the above salt in distilled water and rectified spirit of wine*. I should observe that, in these proportions, the part is somewhat corrugated, which is not the case if one-third of the saline solution be used with two of spirit; yet, in the former quantities, I have some reason to think the appearances of disease may be more securely preserved."

This solution, he says, seems to have the property of fixing the blood in the extreme ramifications, without constringing the vessels themselves; while rectified spirit corrugating the delicate membranes of the minutest vessels, repels their contents into the larger, the thicker coats of which are easily acted on, and thus reduces the appearances of inflammation, &c.—*Lon. Med. Gaz. Dec.* 1828.

---

16.—*Danger of Artificial Inflation of the Lungs.*—The practice of artificial inflation of the lungs, as a means of recovery from drowning, has been objected to before the Academie des Sciences, on the strength of experiments made by M. Leroy d'Etortes, on various animals, especially on Sheep, which are stated to prove, that the practice is attended with great danger, and that a strong inflation is capable of producing instant death, although some animals are better able to bear the process than others, a dog, for instance, than a sheep, on account of the stronger texture of the lungs. The experimentalist infers, that the number of persons restored to life from drowning, is less than it would be, but for the use of inflation as a remedy for their recovery.—*Bos. Med. and Surg. Jour.*

---

17.—*Iodine for Chilblains.*—The tincture of Iodine has been recommended for the cure of chilblains. Two or three applications of it are said to restore the skin to its natural state.—*Ib.*

---

18.—*Sting of a Wasp.*—The editor of a French Journal says, the root of the onion, or of garlic, bruised, aud applied over the part stung by the wasp, instantly subdues the pain. The spirit of hartshorn and soap-lees have the same effect.—*Ib.*

---

19.—*Success of cold effusions in Hysteria.* By Dr. Levacher. The patient, æt. 15, after a paroxysm of anger, was attacked with all the symptoms characterizing hysteria in its most violent form, which continued with undiminished severity for the space of four days, notwithstanding the employment of leeches, sinapisms, the warm bath, enemata, and a variety of antispasmodics. Dr. Levacher ultimately directed cold well water to be poured upon the head from the height of two feet and a half, and permitted to run down over the loins. This affusion was followed by an immediate mitigation of all the symptoms. She rested well during the night, and the next morning her pulse was regular, and the hypogastrium no longer painful. This favorable condition, however, was interrupted towards noon by a fright which she received from a dream that robbers were breaking into her room: she awoke in great terror, and a repetition of the convulsions was the consequence. Recourse was again had to the cold affusions, and with the same salutary effects as before.—The day following, she was still better, but the headache continuing, a bladder filled with iced water was applied to the head, and bottles of warm water to the feet, which succeeded in removing it. Two days later she had a return of the convulsions, but they speedily yielded to the foregoing plan, and did not again recur.—*Journal des Progres, &c.*

---

20.—*Radical cure of a Hydrocele, by the introduction of a needle into the Tunica Vaginalis.*—The patient had for several years been incommoded by a very large hydrocele, which had been repeatedly punctured and injected, but without success. The fluid was constantly reproduced, and the patient was desirous of undergoing an operation which would effect a radical cure. The following plan was adopted by Dr. Moro. The patient being seated, a trocar was introduced at the usual place of puncture; the preforator was then withdrawn, leaving the canula, and through the latter a long needle, like those used in acupuncturation, was introduced from below, upwards in the direction of the abdominal ring ,and near the spermatic cord, so as to pierce the integuments on the side of the penis. After the water had been entirely discharged, the canula was withdrawn,and the two extremities of the needle approximated and twisted together. A slight degree of pain and heat in the groin,which was propagated towards the kidneys, followed the operation, but disappeared in the space of twenty-four hours; six days afterwards, the scrotum had regained its natural form and dimensions; two red points were seen where the integuments had been perforated by the needle. The latter was divided by the cutting pincers, and removed without the slightest difficulty. A year and a half have now elapsed, and there has been no return of the disease.

*Annali Univ. di Medicina.*

---

21.*Nux Vomica in Chronic Diarrhœa and Intestinal Hemorrhage.*—M. Recamier, of the Hotel Dieu, having learnt that the nux vomica was used in chronic diarrhœa by the practitioners of the north, administered it in the following case, with success.

A man, fifty years of age, eminently nervous, had been long subject to alternations of bilious diarrhœa and intestinal hemorrhagy, which had reduced him to an alarming state; his lips and countenance were pallid. Sometimes the bilious flux preceded the hemorrhoidal discharge; at others the order was reversed. Colombo, semirouba, and powdered charcoal, had been tried without effect. Opium in the dose of a quarter of a grain, disagreed. One-eighth of a grain of the alcoholic extract of nux vomica was then prescribed. On the following day the stools were reduced from twelve or fifteen to three or four. The dose was then doubled, one quarter of a grain given, and the patient was speedily cured by this treatment.—*La Clinique.*

---

22.—*Diagnostic Symptoms of Dislocation of the Head of the Femur into the Ischiatic Notch. By Dr.Buchanan.**—Sir A. Cooper has observed, that "of all the dislocations of the thigh, this is the most difficult to detect, because the length of the limb is but little altered, and the change in the position of the knee and foot is not so marked as in the dislocation upwards." The symptoms which Sir Astley details as diagnostic are the following.

"The limb is from half an inch to one inch shorter than the sound one, but rarely more than half an inch. The natural projection formed by the trochanter major is diminished, and is inclined towards the acetabulum, but still remains at right angles with the ilium. The head of the bone can only be felt in very thin persons, and then not very distinctly. The knee and foot are turned inwards, and the great toe rests against the ball of the great toe of the sound limb. When the patient is erect, the toe touches the ground, but the heel does not quite reach it, and the knee is bent, and projects a

---

* Glasgow Medical Journal, No. IV.

little forwards. The motions of the joint are, in a great degree, prevented, admitting of but slight flexion and rotation."

These symptoms, Dr. Buchanan considers as characteristic of dislocation, when it actually exists, but simulated so far by other affections of the joint, as to lead to error when adopted in every instance, as our guide. A truly diagnostic sign, then, being wanted, the author of the paper believes that he has found it, and proceeds to make it known. In the summer of 1821, a young woman was brought into the Glasgow Infirmary, after falling from a height, a good deal bruised and affected, soon afterwards, with a paralysis of the right leg. These symptoms had nearly disappeared when Dr. Buchanan first saw the case, but others were present closely corresponding with those laid down by Sir Astley Cooper, as marking dislocation into the ischiatic notch; an accident which was thought to have happened to the patient. Gradually, however, the limb re-assumed its natural aspect, and showed that the doctors had been taken in.

"In reflecting on the preceding case, it occurred to me, that it would be of much importance in supposed cases of dislocation into the ischiatic notch, to institute a comparison of the limbs, not while resting in the same plane with the body, but when bent to a right angle with the abdomen; the reason of which must be sufficiently apparent, from considering the anatomy of the joint, and the nature of the injury. The head of the femur being thrown almost directly backwards, and very little upward, it is clear, that so long as the limbs remain in the plane of the body, there can be very little difference in their relative length; since that difference is only measured by the extent of the displacement upwards. But if the limbs be slowly bent towards the abdomen, the difference in their length must become greater and greater, till it attain a maximum, when the limbs are at right angles with the body; the luxated limb being then shortened by the whole extent of the dislocation backward. I had no opportunity of verifying this reasoning by actual observation, till the occurrence of the following case, in 1826, and I had then the satisfaction to find, that the mode of examination described above, yielded the only symptoms which were perfectly unequivocal."

A child, three years of age, fell from a chair, and when seen by Dr. B. six days after the accident, presented the following appearance. The girl could not walk nor stand without assistance, but, when supported by her mother on the sound limb, the toes alone of the other touched the ground, were a little turned in, and rested in the same transverse line with the metatarsal bones of the opposite foot. The knee was considerably bent, and advanced before its fellow; the shortening of the limb, if any existed, could not have been considerable; the thigh could be bent towards the abdomen with facility, and seemingly without producing much pain, whilst abduction and rotation were not only painful, but comparatively difficult; the head of the femur could not be discovered; the trochanter was little displaced; the contour of the haunch not materially altered; and, lastly, the limb altogether had more the appearance of being drawn up by a voluntary effort, than of being actually shortened by displacement. These symptoms were dubious, but uncertainty vanished the instant the patient was placed upon her back upon a table, and both thighs bent to a right angle with the trunk of the body. The shortening immediately became most decided, the affected knee, resting in the hollow below the condyles of the opposite limb. The trochanter, also, was felt much farther back than on the sound side.

Dr. Watson, Dr. Young, and Dr. Auchincloss, being also satisfied of the nature of the accident, the reduction was effected next day, by placing the patient in a sitting position on a table, to which she was fastened down by a sheet, and secured by a man standing astride it behind her—performing extension by a bandage round the ancle—and lifting the head of the femur over the edge of the acetabulum, by means of a handkerchief passed under the thigh. The reduction took place with an audible snap.

This mode of examination, our author considers applicable to dislocation into the foramen ovale, but there is just this slight objection which strikes us, namely, that one of the distinguishing marks of these dislocations is the immobility, at any rate impaired mobility, of the limb. In the dislocation into the ischiatic notch, Sir Astley Cooper expressly states, that the thigh admits of but "slight flexion and rotation," and when the displacement is into the foramen ovale, we should think the want of motion would be greater still. In some cases, however, the one for example detailed by Dr. B. the limb was so mobile, as to admit of the application of the test, and, in such, it may prove a valuable addition to our diagnostic marks. In cases of contusion, or inflammation of the joint, simulating dislocation, such an examination seems perfectly applicable.

*Med. Chir. Review.*

---

23.—*Traumatic Tetanus—Pathology of.*—We are inclined to think, that if ever the true pathology of traumatic tetanus be discovered, it will be found, that the nerves leading from the injured part will exhibit symptoms of inflammation, and that this inflammation will extend to the spinal marrow. In a former number* we alluded to some researches made by M. Lepelletier, surgeon of the Hopital du Mans, on the subject of tetanus traumaticus. He had scarcely published his paper, when the following interesting case occurred, confirmatory of this gentleman's doctrines.

*Case.* Peter Crepon, aged 15 years, feeble in constitution, and miserable in circumstances, had been effected with a tumour on the *left* heel, from the age of three years, which, gradually increasing, ulcerated, assumed a malignant character, and, finally led to amputation, at the Hospital of Mans, on the 28th of December, 1827.

---

* See No XVI, April, 1828, p 439.

Nothing particular occurred till the 5th of January, 1828, when he complained of convulsive twitchings in the stump. On the 6th, symptoms of tetanus came on and were soon unequivocal. It was remarked, that the temporal and masseter muscles of the *left* side only were affected with trismus. This corresponded with the amputated limb. We shall not detail the daily treatment and symptoms. Repeated venesections were performed, and, in almost every instance, they relieved the tetanus for a time; but nothing could arrest the progress of the disease, and death took place on the 9th of January.

*Dissection.* A small depot of purulent matter was found in the stump, on the head of the fibula, with traces of inflammation in the neighborhood. The sciatic nerve at this place was found so much injected as to be of a violet hue, while the same nerve, in the other limb, was of its natural color. This discolouration continued, with some interruptions, up to the hip. The patient, during life, complained of pain in the track of this nerve, when pressed by the hand—and that for some days previous to the development of the trismus. The discoloration could be traced in some branches of the sciatic nerve distributed to the crural muscles. The pulpy substance of the nerve was found to be somewhat softened. The pia mater of the spinal marrow was decidedly injected; especially towards the middle of the dorsal region, where the medulla itself was softened and degenerated in structure. Some reddish effusion was also found under the membranes of the medulla spinalis.—*Jour. de Progres.*

The narrator thinks that the evidence of the inflammation of the nerve from the stump to the spine, as the cause of the tetanus, is here complete. We do not think that the deduction can be denied, as far as this case is concerned; but, unfortunately, there are other cases of traumatic tetanus, where the most careful dissection cannot detect any inflammation of nerve between the seat of injury and the encephalon. A case occurred at St. George's Hospital, where the spinal marrow, and the nerves of the limb were carefully examined, but without success. These conflicting facts prove that tetanus may be induced by the local injury, with or *without* evident change in the spinal marrow or nerves.—*Med. Chir. Rev.*

---

24.—*Ointment for Glandular Swellings.*—Professor Dupuytren recommends the following as a formula for an ointment to be rubbed over the region of the engorgement. The stimulating action of the ammonia aids, he thinks, very powerfully the deobstruent property of the mercurial ointment. Take *strong mercurial ointment,* twenty four parts; *hydrochlorate* (muriate) *of ammonia,* six parts. Mix accurately.—*N. A. Med. and Surg. Journal.*

---

25.—*Caustic Paste.*—Professor Graefe recommends, as a preparation adapted to destroy the callosities accompanying fistulas, *perchloride of mercury* (corrosive sublimate) two drachms; *gum arabic*

and distilled water, of each twenty-four grains. Mix them with care. The paste is to be applied to the callous parts.—*Ibid.*

---

26.—*Collyrium Siccum for the Opacity of the Cornea.*—Take *red oxide of mercury* and *white agaric* of each half a drachm; *white sugar*, one ounce. Mix accurately so as to make a very fine powder. A small quantity is to be blown on the eye daily.—*Ibid.*

---

27.—*The Effects of entire Abstinence from Solid and Liquid Food.*—This is a question which exercises a very important bearing on therapeutics. It has been the theme of much, and at times no very measured, discussion. It is one, moreover, which nearly and deeply concerns mankind at large, not only on account of abstinence being made a means of relief from disease by the professional attendant, but also on account of the possible widely extended application of the measure among all classes of society, without any other counsel than the consciousness of their being sick. The plainest dictates of instinct, and arguments of common sense, are in favor of its adoption under circumstances of bodily disturbance and disease; and it is to be greatly regretted that false theory and the vaguest analogies should have induced a portion of the medical community to discountenance it.

We wish at this time more especially to direct the attention of our readers to the *Experimental Inquiries* made by Dr. Collard de Martigny—1. *On the effects of complete abstinence from solid and liquid food on the organs of the body; on the quantity and composition of blood, and the nature and course of the lymph:* 2. *On the morbid alteration of the fluids; the proper time for abstinence and bleeding in certain diseases; the use of the lymphatic vessels; and the existence of a lymphatic temperament.*

The effects of entire abstinence on the organs, as evinced in dogs and rabbits starved to death, are general and excessive emaciation, and a diminished size and colourless state of the muscles. The heart has little volume, and, as well as the aorta, vena cava, and pulmonary artery, contains a very small quantity of blood. The lungs are entirely empty of blood; the lining mucous membrane is pale; the liver, spleen, pancreas, and kidneys are remarkably pale and small; their vessels empty, and parenchyma having little consistence. The vesical mucous surface is of a pale white. The gall bladder is of very considerable size, and distended with a large quantity of greenish yellow bile, limpid and very fluid.

The stomach is contracted on itself, the mucous coat exhibiting numerous folds, running in every direction, but especially from the cardia to the pylorus. The medium thickness of the stomach is a line and a half; its cavity is empty; the pyloric portion has a deep yellowish tint; when washed, its internal membrane towards the cardia is of a silvery whiteness, as Haller has already remarked, and towards the pylorus of a yellowish white. *In no part did it exhibit the slightest trace of inflammatory alteration.*

Throughout the entire extent of the intestinal tube the mucous memberane is wrinkled; that of the small intestine is deeply coloured with a greenish yellow, which is less evident in the large intestines. *As in the case of the mucous membrane of the stomach, that of the intestines exhibited, in its appearanae, color, aad consistence, no sign of inflammation.* Both the small and large intestines are of diminished diameter, and contain a greenish yellow matter; which, fluid in the duodenum, increases in consistence in proportion as we leave the stomach.

In one case, the intestinal tube contained, throughout its entire extent, a small quantity of a dark green matter of a very putrid odour. The mucous surface of the inferior portion of the small intestine, and of the cœcum, in which it most abounded, was studded with violaceous spots. The mucous membrane of the stomach was strongly contracted and wrinkled, and of a slightly roseate hue at its pyloric extremity.

There is a notable difference in the duration of life between dogs and rabbits deprived of all nutriment; the carnivorous animals surviving much longer than the herbivorous. Age and size are also modifying conditions; since the older and larger the animal, the greater are its powers of living under a privation of all aliment.—Thus, of three dogs submitted to the above experiments, the largest lived thirty-two days; the second, which was larger than the third, lived twenty-seven; whilst the third and smallest survived only twenty to twenty-one days.

The medium period in which three rabbits survived entire abstinence from food was ten to twelve days. It is greatly abridged if the animal be very young; as in the instance of three rabbits six weeks old, two died on the seventh, and one on the eighth day of starvation. A rabbit only three weeks old, died on the night succeeding the third day of privation from food.

The contrast in the powers of enduring hunger between the carnivorous and herbivorous animals, was clearly exhibited in the cases of the rabbits seven months old, and the third dog mentioned above. Their size was nearly equal, and yet the first lived but ten or twelve days, while the latter survived three weeks.

In rabbits there is much less general emaciation and loss of the cellular tissue of the parenchyma of the organ; the muscles preserve nearly three-sevenths of ther first size. *All the mucous membranes are white and without any appearance of inflammation.* The cavities of the heart, the beginning of the aorta, and venæ cavæ and pulmonary vein, contain a small quantity of blood. The small vessels and capillaries are destitute of this fluid; the cornea is sunken and rather cloudy. It was never observed to be ulcerated.

In regard to the *effects of abstinence on the quantity of the blood,* Dr. De Martigny shows, by direct experiments, that there is a progressive diminution of this fluid throughout the entire period of alimentary privation.

Not only is there an *alteration in the quantity*, but also in the quality or composition of the blood after prolonged abstinence.—The quantity of albumen is increased, that of fibrin diminished. For farther particulars on this head we refer to our department of chemistry; wishing on the present occasion to restrict ourselves to what has a direct therapeutical application in the experiments of Dr. De Martigny.

This gentleman concludes his chapter on "*the effects of entire abstinence on the quantity and course of the lymph,*" by saying that the velocity and course of the lymph in the vessels must decrease in direct proportion to the diminution in quantity of this fluid: and that, towards the conclusion of the period of forced abstinence, the slowness of the course of the lymph is such, that this fluid merely flows in drops, at intervals, from an opening made in the thoracic duct above the diaphragm. The first and immediate effect, however, of fasting is to accelerate the progress of this fluid. The change in the *composition of the lymph* after entire and prolonged abstinence is also distinctly shown by the author.

He asserts, in reference to the evidences of local inflammation, designated in some of the examinations after death from abstinence, that these are a complication, and are at any rate of very rare occurrence; since out of eighteen animals opened and examined with the most scrupulous accuracy, he only met with three examples of this nature. Two of these had an evident origin. In the first, the microscopic ulcerations of the tracheal membrane were consecutive to an incision of the trachea, and probably also to the accidental introduction of dust or other light matters floating in the air. The violaceous spots of intestinal mucous membrane of the dog already mentioned, were due to the presence of excremental matters, become putrid and infectious in the intestine.

In the recapitulation of his subject, the author says that the *disease* produced by prolonged abstinence until death, is the result, 1st, of an alteration of the solids; 2d, and chiefly of alterations of the blood, which are at once the *mediate* and *immediate* causes.

The "*reflections on the proper time of low diet and bleeding in certain dieases,*" without being long or profound, are nevertheless worthy of all attention. Abstinence has long been considered to exert the same effects which are produced by blood-letting, but in a slower manner. The author thinks that we rely too much on the safety with which low diet can be persevered in. What, he asks, results from this course? "The patient continually loses flesh; his sensibility and imagination are exalted, while his physical and moral powers are prostrated; his sleeplessness is increased; his weakness or concentration of pulse, paleness of the mucous surfaces, and diminution of secretions, announce the alteration in the quantity and composition of the blood. These are identical effects with those of general bleeding. Why then bring about slowly a state of things which we should tremble at causing with greater promptitude?" It might be said in reply that it is not these effects which the physician deprecates, so much as a sudden general prostration and loss of balance between the heart and capillaries, which are occasionally caused by injudicious general bleeding.

The author next adverts to the exalted sensibility of the digestive canal, caused by long withholding food, and the consequent inability, after a while, to tolerate it in a common or even reduced quantity.

A caution is given to the surgeon not to be too prodigal of the blood of a patient suffering from wounds or fractures, since it has been seen that the wounds of animals subjected to prolonged abstinence cicatrize very imperfectly.

A rigid diet is more especially of dangerous tendency in cases in which there is a purulent cavity in any part of the body, or a suppurating wound of a bad character. The first effects of fasting are, as already stated, a diminution in the quantity of blood and an increase in that of the lymph; that is to say, a very accelerated absorption—of course an absorption of the foreign and purulent matters from the sources above indicated. Among numerous other examples, the author cites the cases given by M. Andral; in one of which a woman with chronic nephritis, the thoracic canal was found full of pus, and, some time before death, there had been *marasmus* and *hectic* fever; in two other women dead of *cancer* of the uterus, and the pelves of whom contained putrid matter and many cancerous masses, the thoracic canal held a puriform fluid and a matter analagous to the cancerous masses. Instances are recorded by M. M. Vallerand de la Fosse, Velpeau, and Bouillaud, of encephaloid and cancerous matters in the veins. Dupuytren and Magendie furnish similar cases.

The two last chapters of the long and interesting paper of Doctor Collard de Martigny, are on the *functions of the lymphatic system*, and on the temperament characterised by the predominance of this system. These have been noticed in their proper place, under the physiological head.—*Ibid.*

---

28.—*Of the Medicinal properties of Leontodon Taraxacum.*—Mr. Houlton, in the London Medical and Surgical Journal for September, after adverting to the great discrepancies of opinion in regard to the dandelion, thinks that the cause is to be found in the different seasons in which the extract of the juice is prepared, and the corresponding variety in the activity of that sold in the shops. As usually obtained from the latter, it has very little virtue.

The most uniform and active preparations of this plant may, in Mr. H's. opinion, be obtained by carefully evaporating spontaneously the expressed juice of the roots taken up in August and September. In cases of chronic disorder of the digestive organs not produced by intemperance, its efficacy is frequently very decided. In visceral derangements from intemperance, Mr. Houlton did not find it of much service, but in females and other persons of sober habits, and of studious and sedentary pursuits, it has been very beneficial, increasing the flow of bile and allaying that uneasiness which the dyspeptic frequently experience about the hepatic region.—*Ibid.*

